# Audio Augmentation for Non-Native Children’s Speech Recognition through Discriminative Learning

**DOI:** 10.3390/e24101490

**Published:** 2022-10-19

**Authors:** Kodali Radha, Mohan Bansal

**Affiliations:** School of Electronics Engineering, VIT-AP University, Amaravati 522237, India

**Keywords:** non-native children speech recognition, data augmentation, speed perturbation, discriminative models, mutual information

## Abstract

Automatic speech recognition (ASR) in children is a rapidly evolving field, as children become more accustomed to interacting with virtual assistants, such as Amazon Echo, Cortana, and other smart speakers, and it has advanced the human–computer interaction in recent generations. Furthermore, non-native children are observed to exhibit a diverse range of reading errors during second language (L2) acquisition, such as lexical disfluency, hesitations, intra-word switching, and word repetitions, which are not yet addressed, resulting in ASR’s struggle to recognize non-native children’s speech. The main objective of this study is to develop a non-native children’s speech recognition system on top of feature-space discriminative models, such as feature-space maximum mutual information (fMMI) and boosted feature-space maximum mutual information (fbMMI). Harnessing the collaborative power of speed perturbation-based data augmentation on the original children’s speech corpora yields an effective performance. The corpus focuses on different speaking styles of children, together with read speech and spontaneous speech, in order to investigate the impact of non-native children’s L2 speaking proficiency on speech recognition systems. The experiments revealed that feature-space MMI models with steadily increasing speed perturbation factors outperform traditional ASR baseline models.

## 1. Introduction

Automatic speech recognition (ASR) technology for adults has become popular in many applications owing to the accessibility of large volumes of data and substantial computational capabilities, and new studies have already shown that ASR systems can accomplish quality standards similar to human transcriptions for some tasks [1]. However, ASRs proved their ability to recognize English spoken by adults or children with a native accent. They, on the other hand, continue to struggle due to the coexisting variability of non-native and children’s speaking characteristics, which include the following:

- A lack of accessibility to a large amount of training data for non-native children’s speech [2];
- Reading miscues, such as lexical disfluency, hesitations, intra-word switching (combining two languages within a single word), incomplete words, and filled interruptions, word repetitions, word boundary errors, and the adoption of some native language sounds and phonology are among them.

Because of its prominence as the language of formal instruction, English has become a significant mode of communication among Indian children [3]. However, the aforementioned reading miscues can significantly degrade ASR efficacy [4,5,6]. Therefore, ASR systems should continue to expand in order to more effectively process children’s speech from a non-native population. The key application field is the artificial assessment of L2 speaking proficiency, where ASR difficulties are exacerbated by the speakers with low levels of speaking ability, particularly for children. One of the similar applications available now for detecting and correcting pronunciation errors is “SPIRE-fluent”, a self-learning application for teaching oral fluency to L2 English learners [7]. However, the targeted users are class IX and above, job-seeking graduates, etc. ASR technology developed by “SOAPBOX” labs is being used to assist kids by transcribing their native accent as they read a story. This transcription is then compared to the text of the reading passage. The reliability of the student’s reading attempt is then shown by the fluency algorithm [8].

This encourages us to focus the research on non-native children, where most of the children’s speech corpora are costly and restricted in terms of accessibility. On the other hand, a considerable quantity of Chinese speaking English data (e.g., SpeechOcean [9]) are publicly available. Moreover, a good proportion of the datasets for Indian L2 speakers are limited. As a result, the proposed work involved the collection of speech corpora of different voice tasks from children and made publicly available. Speed perturbation-based data augmentation is being investigated to deal with the lack of training data from Indian children speaking English. To better address the aforementioned gaps, the current state-of-the-art ASR can act as a forerunner for non-native children’s speech.

This article’s details are as follows: Section 2 provides a brief overview of the existing non-native children speech recognition using several state-of-the-art models. Section 3 describes the details of the collected children’s speech database, which is followed by the proposed state-of-the-art models, including the mathematical intuition of the discriminative models in Section 4. The experimental procedure that addresses the corpus setup and artificial data creation used in the study is presented in Section 5, and the experimental evaluations for different styles of children’s speech are reported in Section 6. Finally, Section 7 outlines the conclusion and the future expansion of the proposed work.

## 2. Related Work

Children’s automatic speech recognition is a subject of great interest these days, as they are more comfortable interacting with digital personal assistants, such as Amazon Alexa, Microsoft Cortana, and Google Home. Children will advantage the most from technologies, such as automated reading assessment [10] and an interactive reading tutor [11], assisting them to learn both their first and second languages with minimal support from instructors. Another application is accent classification from native and non-native UK children’s speech, using reliable cues extracted from five–year-old pre-school children’s speech [12]. Another use in children’s speech is the classification of para-linguistic speech cues for adults and children using a phoneme-based model [13,14]. Since 2020, Interspeech has launched a challenge [15] to improve research on non-native children’s speech recognition technology, which is still failing due to immature vocal tracts, disfluencies [16], ungrammatical syntactic structure due to their L2 proficiency [17,18], use of out-of-vocabulary words (OOV) [19], and often due to a lack of publicly accessible data.

Many efforts are underway towards enhancing the efficiency of non-native children’s speech recognition, including data augmentation, the robust extraction of feature techniques, transfer learning, multi-task learning, and so on. Children aged 11 to 15 years old across Arab countries, China, France, and Germany obtained a word error rate (WER) of 13.4% employing bidirectional long short-term memory (BiLSTM)–recurrent neural network (RNN) models with read speech, picture narration, and spontaneous speech [20]. An investigation on transfer learning with Italian, German, English, and Swedish children aged between 9 and 10 years using a deep neural network (DNN) model achieved a WER of 14.2% for Italians speaking English and 15% for German children speaking English [18]. Additionally, prosody and spectrogram-based data augmentation are employed on the Trentino Language Testing (TLT) school non-native Italian children’s speech corpus, aged 9–16 years, on read speech incorporating factored time-delay neural networks (TDNN-F) and a convolution neural network (CNN) along with BiLSTM and vocal-tract-length normalization (VTLN) and attained a WER of 18.71% [21]. Consequently, the Mel-frequency cepstral coefficient (MFCC) and i-vectors are extracted and then speed and spectrogram perturbed to boost the effectiveness of the factored time-delay neural networks and convolution neural network (CNN-TDNN-F), resulting in a relative improvement (RI) of 17.76% over the same TLT corpus [22]. Furthermore, using spectrogram augmentation with a TDNN-F and LSTM on the TLT Italian children speech corpus [23], an RI of 17.87% is achieved compared to [21] and a 10.7% RI compared to [22]. Moreover, all available children’s speech corpora, such as OGI kids, MyST, CU, and CMU kids, ages 5 to 16, augmented the data with speed perturbation, room impulse response (RIR), babble noise, and non-speech noise, were incorporated on the CNN-TDNN-F model to achieve a WER of 16.59% using the minimum Bayes-risk (MBR) decoding technique [24]. Finally, the CNN-TDNN-F and Esp-Net models were used to investigate with pitch, speed, tempo, volume, and reverberation perturbations on a corpus of Mandarin children’s speech-language therapy (SLT) with read and conversational speech for 4–16 year old children, yielding a character error rate (CER) of 16.48% [25].

Neural networks struggle with data scarcity due to the large number of parameters more frequently than conventional methods, especially in low-data scenarios. As a result, the proposed work is significant for children aged 7–12 years and employs feature-space discriminatively trained models on various methods of speeding up data augmentation.

## 3. Children Speech Corpora

A collection of non-native children’s speech was developed because there was no publicly available corpus of non-native children’s speech suitable for the planned analysis at the time of authoring. As a result, data collection is required and the procedure for it will be discussed further below.

### 3.1. Participants

A total of 20 children with a gender composition of 11 females and 9 males in concrete-operational stage [26] with an age group between 7 and 12 years is considered for data collection. All the children are non-native English speakers and are bilingual, i.e., they speak Telugu, an Indian regional language, alongside English. At school, all the students speak English with age-appropriate proficiency. All the children and the parents gave their approval for participation in the research.

### 3.2. Data Collection

In order to assess children’s pronunciation and communication skills, the sentence repeating task and the picture narration task were devised in data collection. As it is targeted for primary and lower middle class students around the ages of 7–12 years, simple sentences and pictures were used. In “read speech” task, children repeat the sentences which were framed as a part of daily routine (e.g.,: *I will wake up early in the morning and pray to God*) to measure the phonetic ability. To evaluate working memory and attention, spoken digits from 1 to 15 were included but without repetitions or mistakes. To make the recording setting child friendly, few most well-known rhymes were also added, such as “rain rain go away, come again on other day”. Each sentence lasts for 2–3 s duration.

In the picture narration task, children are expected to describe the black and white picture of objects, such as pencil, umbrella, cap, etc., which are available in SurveyLex cloud [27]. The ability of non-native children to speak English fluently and effectively can be evaluated using this method of speech and is known as “spontaneous speech”. This activity has the potential to represent children’s higher-order thought associations, vocabulary, attentiveness, alongside disfluencies, false starts, pause lengths, and other issues. The length of the utterance varies depending on how well the children can communicate in English.

### 3.3. Data Processing

The open source *SurveyLex* [28] platform was deployed in the device at a distance of 2 feet from the children to record speech. Students are allowed to sit in front of a computer in a relaxed pace. All the audio files were recorded in *.wav* format that can use a stereo channel at a sampling rate of 44.1 KHz and a bit rate of 16 bits per sample. To analyze the variability in words and sentences, each survey is taken as many times as the child can, up to a maximum of 10 times per child. A total of 199.2 min (3.32 h) of data has been recorded. The audio files of children in English were recorded during class days, because robotic and computer applications are required to cope relatively well in classroom (i.e., real-world) environments which can often be noisy [29]. As a result, the planned corpus is recorded in this natural setting that involve doors closing, bell ringing, and other children talking sounds in neighboring rooms. A realistic representation of a minimal practical noise level in natural scenario is acceptable, which allows us to evaluate recognition accuracy with greater veracity.

Both read and spontaneous speech data were carefully transcribed, and all raw audio files, as well as transcription, may be found at Non-Native Children Speech Mini Corpus [30].

## 4. Proposed System Overview

**Figure 1:**
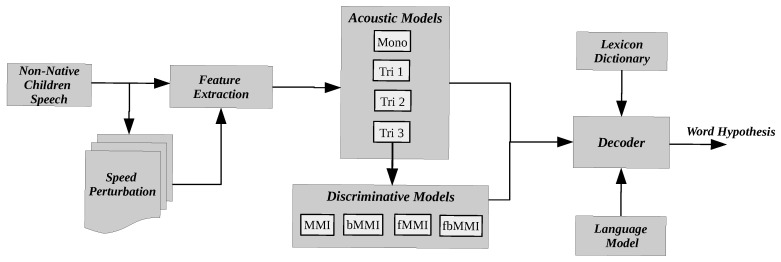
Proposed discriminatively trained non-native children speech recognition using speed perturbation-based audio augmentation.

The collected children’s speech is a stereo channel at a sampling rate of 44.1 KHz, which is down-sampled to 16KHz rate with single channel using *Sox* [31]. Initially, the proposed model is trained with re-sampled children speech and further experimented using speed perturbation-based data augmentation in order to handle the data scarcity as demonstrated in Figure 1. The most popular front-end feature extraction, MFCC, has been employed with frame length of 25 ms and an overlap of 10 ms after successful adaption of cepstral mean and variance normalization (CMVN).

The work is usually composed of two parts: acoustic modeling (AM) and language modeling (LM), with one focusing on mapping a set of acoustic feature vectors to suitable phonetics and the other subsequently transducing a set of phonetic units into proper sentences. The baseline data without augmentation and the augmented feature vectors are initially trained using acoustic models, such as context-independent mono-phone model (mono) and context-dependent triphone models (tri1, tri2) with delta and double-delta features on top of MFCCs. The linear discriminant analysis with maximum likelihood linear transformation (LDA-MLLT) (tri3) is being used to further transform the feature dimension, which are aligned using feature-space maximum likelihood linear regression (fMLLR). To reduce the significant deviation among word representations in train and test models, the output of tri3 is fed to discriminative models, such as maximum mutual information (MMI), boosted MMI (bMMI), feature-space MMI (fMMI), and boosted feature-space MMI (fbMMI), as shown in Figure 1.

For language modeling, conventional 3-gram language models (LM) [32] in the form of weighted finite-state transducers (WFST) [33] are commonly used, whereas Speech Technology and Research Laboratory (SRILM) is an open-source language modeling toolkit with remarkable features that is used to rescore the acoustic model outputs. Furthermore, test data are used to evaluate the performance of the proposed model, and the decoder results are depicted in terms of word error rate (WER).

### 4.1. Acoustic Models

For decades in ASR engines, Mel-frequency cepstral coefficients (MFCC) have been the most extensively employed acoustic feature vectors. A dense representation of the data is created by extracting MFCC feature vectors from raw data with a frame size of 25 ms. There has been a lot of research on extracting features using MFCC due to its ability to simplify the speech amplitude spectrum in a cosine form on a non-linear Mel scale. The primary goal of speech recognition is to find the best possible sequence of words from raw audio input using a language model. Acoustic observations are indicated as *X*, and a sequence of words is represented by $W_{r}$, in order to form an acoustic model as shown in Equation (1)

(1) $$\tilde{W_{r}}=\underset{W}{\mathrm{argmax}}P\left(X\right|W_{r})P\left(W_{r}\right),$$

where $\tilde{W_{r}}$ is the word hypothesis, $P\left(X\right|W_{r})$ is the acoustic model, and $P(W_{r})$ is the language model.

A Gaussian mixture model (GMM) may be used to estimate the distribution of phone features. The hidden Markov model (HMM) may be used to model the transition between phones and associated observations. The HMM model is comprised of hidden states and observables, and the Baum–Welch method [34] can build the HMM model provided all the training set. The Viterbi algorithm is used to determine the most probable sequence of hidden states.

Finally, two important metrics were used to determine optimal system performance [35]: the word error rate (WER), which is equal to the number of substitutions (S), insertions (I), and deletions (D) divided by total number of words in the actual utterance (N) and is given in Equation (2)

(2) $$\%WER=\frac{S+D+I}{N}\times 100$$

and the percentage relative improvement (%RI) is predicated on the division of absolute increment corresponding to new values (N) by their original values (O) as shown in Equation (3)

(3) $$\%RI=\frac{\left(N-O\right)}{O}\times 100$$

### 4.2. Discriminative Models

The ultimate goal of discriminative modeling is to enhance the quality of ASR by properly training the statistical parameters of HMM. Although there are several others, MMI is one of the most extensively used discriminative techniques. Despite the fact that several researchers have reported significant improvements in native adult and child speech recognition using discriminative models [36,37,38], persistent improvements in non-native child speech recognition using discriminative models are still elusive. The key contribution of this article is the use of feature-space MMI and boosted feature-space MMI, which reliably outperform MMI and boosted MMI and are likely to be effective methods for discriminative training.

#### 4.2.1. Maximum Mutual Information (MMI)

MMI aims at maximizing the posterior distribution, or likelihood of data, for certain right-word patterns while lowering the total likelihood of data for all possible word sequences in the lexicon. In a nutshell, the objective function of MMI is developed by finding the correct word hypothesis more likely whilst making the incorrect word hypothesis less likely [39,40]. The MMI objective function is achieved by optimizing mutual information on a sequence of observations *X*, which requires setting the parameter to λ, while the correct word transcription $W_{r}$ is corresponding to HMM of the $r^{\mathrm{th}}$ utterance is derived as shown in Equation (4),

(4) $$\begin{array}{cc} {ϝ}_{\mathrm{MMI}}\left(\lambda \right) & =\sum_{r=1}^{R}\log\frac{P(X_{r},W_{r})}{P\left(X_{r}\right)P\left(W_{r}\right)}\\ \; & =\sum_{r=1}^{R}\left[\log\frac{P(X_{r},W_{r})}{P(X_{r})}-\log P(W_{r})\right]\\ \; & =\sum_{r=1}^{R}\left[\log\frac{P_{\lambda }\left(X_{r}\right|W_{r}{)}^{k}P\left(W_{r}\right)}{\sum_{\hat{W}}P_{\lambda }\left(X_{r}\right|\hat{W}{)}^{k}P\left(\hat{W}\right)}-\log P(W_{r})\right], \end{array}$$

where $P(W_{r})$ is the probability of all possible word sequences in the language model and *k* is a scalable fudge factor in correcting the word estimations.

**Figure 2:**
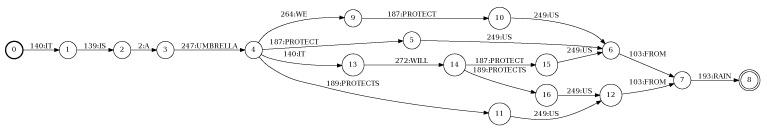
An example word lattice of spontaneous speech utterance: “*It is a umbrella. It protects us from rain*”.

MMI can be further simplified as shown in Equation (5) by setting $P(\hat{W})$ independent of model λ which significantly improves the posterior probability that is possible simply by increasing the likelihood of making a prediction that is identical to hypothesis (numerator lattice) and minimizing the likelihood of acquiring wrong words $\hat{W}$ (denominator lattice). Figure 2 illustrates an example of generated lattice for a possible word hypothesis.

(5) $${ϝ}_{\mathrm{MMI}}\left(\lambda \right)=\underset{\lambda }{\mathrm{argmax}}\sum_{r=1}^{R}\left[\log\frac{P_{\lambda }\left(X_{r}\right|W_{r})P\left(W_{r}\right)}{\sum_{\hat{W}}P_{\lambda }\left(X_{r}\right|\hat{W})P\left(\hat{W}\right)}\right]$$

#### 4.2.2. Boosted Maximum Mutual Information (bMMI)

In reality, MMI has a number of issues, including difficulty in maximizing the objective function, greater computational cost for maximization, and poor adaptation to unknown data. So, the language model is altered by the inclusion of a boosting parameter. This boosting factor, b = 0.05, is believed to enhance the possibility of wrong words ($\hat{W}$), which leads to more confusion [41]. There seems to be a small exponential change in bMMI when compared to MMI, as shown in Equation (6).

(6) $${ϝ}_{\mathrm{bMMI}}\left(\lambda \right)=\underset{\lambda }{\mathrm{argmax}}\sum_{r=1}^{R}\left[\log\frac{P_{\lambda }\left(X_{r}\right|W_{r})P\left(W_{r}\right)}{\sum_{\hat{W}}P_{\lambda }\left(X_{r}\right|\hat{W})P\left(\hat{W}\right)exp\left(-bA\left(W_{r},\hat{W}\right)\right)}\right]$$

where $A(W_{r},\hat{W})$ is the accuracy of word sequence $\hat{W}$ with respect to $W_{r}$; also, each arc in the lattice is generated by subtracting ‘b’ times the accuracy in log-likelihood.

#### 4.2.3. Feature-Space Maximum Mutual Information (fMMI)

The objective function of feature-space discriminative training is similar to MMI, with the exception that feature transformation is optimized using a very high-dimensional feature vector ($f_{\tau }$), along with global matrix *G*, also known as Gaussian posterior. These transformed feature vectors ($f_{\tau }$) are reflected back into the original feature space by adding it to original features $a_{\tau }$ by a linear transformation technique with T:a⟶b given as $b_{\tau }=a_{\tau }+Gf_{\tau }$.

The feature vector transformations are calculated using gradient descent [42] instead of the Baum–Welch technique [34] to maximize the feature-space MMI objective function, as indicated in Equation (7)

(7) $$G_{ij}:=G_{ij}+\alpha_{ij}\frac{\partial }{\partial G_{ij}}{ϝ}_{\mathrm{MMI}}\left(\lambda \right)$$

where $\alpha_{ij}$ is the learning rate, and objective function of fMMI Equation (8) is obtained from Equation (7) as,

(8) $${ϝ}_{\mathrm{fMMI}}\left(\lambda \right)=\frac{\partial {ϝ}_{\mathrm{MMI}}\left(\lambda \right)}{\partial G_{ij}}=\sum_{\tau =1}^{T}\frac{\partial {ϝ}_{\mathrm{MMI}}\left(\lambda \right)}{\partial b_{\tau i}}f_{\tau j}$$

#### 4.2.4. Boosted Feature-Space Maximum Mutual Information (fbMMI)

The training procedure of boosted feature-space MMI (fbMMI) is analogous to fMMI training as already addressed in the above section. Moreover, in fbMMI, the feature transformation is maximized in reference to the bMMI objective function as shown in Equation (9). The learning procedure of the model and statistics acquisition in fbMMI are equivalent to those in fMMI. The significant difference is that while implementing the forward–backward algorithm on the lattices, it is necessary to estimate the raw phone accuracy for each arc in addition to the acoustic and linguistic scores. The objective function of boosted feature-space MMI is given as

(9) $${ϝ}_{\mathrm{fbMMI}}\left(\lambda \right)=\frac{\partial {ϝ}_{\mathrm{bMMI}}\left(\lambda \right)}{\partial G_{ij}}=\sum_{\tau =1}^{T}\frac{\partial {ϝ}_{\mathrm{bMMI}}\left(\lambda \right)}{\partial b_{\tau i}}f_{\tau j}$$

## 5. Experimental Setup

Children’s read speech, spontaneous speech, and a combination of both read and spontaneous speech have been used in the experimental setup of a non-native speech recognition system. For this purpose, discriminatively trained models are developed using the Kaldi toolkit [43]. The following is a more detailed description of children’s speech corpora and speed perturbation-based audio augmentation.

### 5.1. Data Interpretation

As shown in Table 1, the collected speech corpus is categorized into three parts to evaluate the performance of ASR, given different styles of children’s speech. Children’s reading abilities are critical for enhancing comprehension and understanding. Furthermore, non-native children who are acquiring a second language such as English are known to exhibit a variety of reading miscues. Lexical disfluency, hesitations, intra-word switching (combining two languages within a single word), word repetitions, and the adoption of some native language sounds and phonology are among them. Because certain miscues are not addressed in the standard children’s database so far, hence ASR’s struggle to recognize non-native children’s speech. These sub-word transcriptions, as shown in Table 2, are especially beneficial for detecting pronunciation errors and hesitations in non-native children’s speech [44].

The datasets in this study are intended to solve the aforementioned problems of children’s ASR. Therefore, it includes both read and spontaneous speech, with an orthogonal split (i.e., no identical speaker) of 70% for training data and 30% for test data, respectively. The train set of read speech corpus consists of 1.82 h of data from 15 children. The number of utterances is 1585, with total and unique word count of 18,154 and 155, respectively. The 0.65 h of test data consists of 5 speakers with 588 utterances in which total words are 6769 and unique words are 123. Four distinct images are used to collect the spontaneous speech in children, resulting in 0.67 h of training data which includes 569 utterances with 6906 total words and 131 unique words. There are 156 utterances in the 0.18 h of test data, including 1896 total words and 82 unique words in spontaneous speech. As one can see, spontaneous speech has a high word count despite the fact that the number of utterances is limited. Finally, as presented in Table 1, combined speech is composed of both read and spontaneous speech from children, with an orthogonal split of 2.65 h of training data and 0.67 h of test data. The train set contains 2274 utterances with a total of 26,443 words and 256 unique words, whereas the test set included 624 utterances with a total of 7286 words and 183 unique words.

### 5.2. Speed Perturbation

Due to limited available training data, children’s ASR systems do not perform well with non-native accents. To tackle the problem of data scarcity, the suggested approach collects high-quality children’s speech and augments it with speed perturbation. The mathematical intuition behind speed perturbation, which resamples the original data in time domain via *Sox*, is as follows.

If children’s speech is represented in time domain as *S*(*t*), then warping the duration of an audio signal by a factor of β alters the children’s speaking rate to y(t)=S(βt). The spectral domain representation of S(βt) is $\beta^{-1}\hat{S}\left(\beta^{-1}w\right)$. If β<1, the length of the speech signal is scaled back while signal energy raises to higher frequencies, and vice versa.

**Figure 3:**
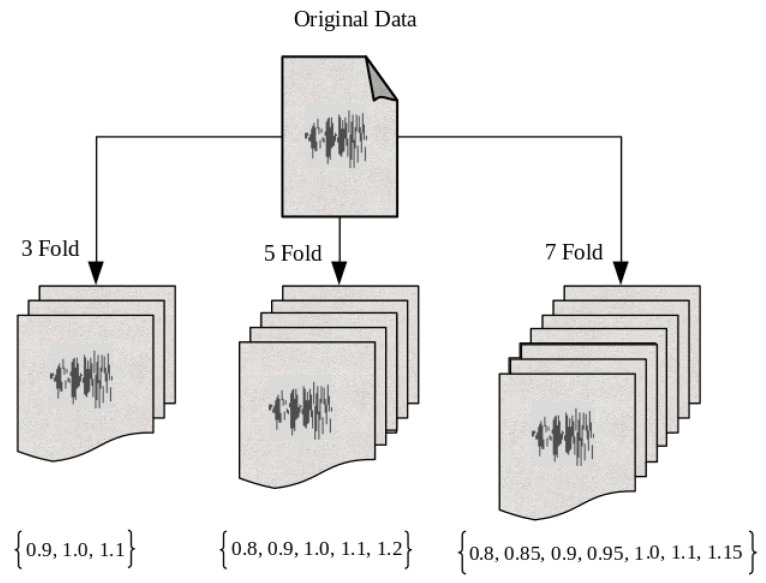
Speed perturbation-based data augmentation.

The experiment is conducted with various folds (1, 3, 5, 7) that stretch or squeeze the duration of speech utterance without influencing the linguistic content of the children’s speech, as shown in Figure 3. The standard Kaldi routine [45] is used to adjust the speed of children’s speech in the proposed work. The baseline data before speed perturbation are interpreted as the Fold-1. By adjusting the speed of the original training data to 90% and 110%, two extra copies of the identical training data were created. This results in a 3-fold training set.

In addition to the standard recipes, 5-fold and 7-fold training sets were developed. With a 5-fold speed perturbation, the speed is changed to 80%, 90%, 110%, and 120% of the original rate, augmenting the training data five times. Similarly, 7-fold speed perturbation data were created by modifying the speed to 80%, 85%, 90%, 95%, 110%, and 115% of the original rate, which is labeled as such in Table 3.

## 6. Experimental Results and Discussion

Considering different styles of children’s speech, several investigations have been carried out by adjusting the speed perturbation factors and examined ASR performance in all the aspects. Initially, acoustic models are being used to train baseline and speed-perturbed data, followed by discriminative models, such as the MMI, boosted MMI, feature-space MMI, and boosted feature-space MMI. This section is a detailed description of the performance evaluation of non-native children’s speech recognition in different speech styles.

### 6.1. Performance Analysis of Proposed Models on Read Speech Task

**Figure 4:**
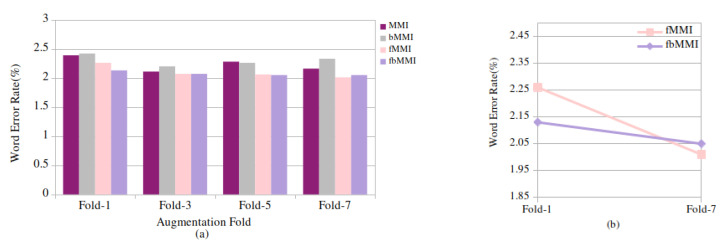
Read speech: (**a**) word error rate (%) for baseline (fold-1) and synthetic (fold-3, 5, 7) data on discriminative techniques and (**b**) (%) relative WER improvement on fMMI, fbMMI for baseline and 7-fold speed-perturbated data.

The GMM-HMM acoustic model is deployed, and it is evaluated using 0.65 h with 588 utterances of baseline children’s read speech data. As shown in Table 4, the initially proposed models are experimented without introducing any speed perturbation factors which are referred to as the baseline results. To conduct monophone (mono) training, 500 total Gaussians were considered, with 1.25 boosting silence. Following, triphone (Tri1) training with 500 tied states (senones) and 2000 cluster leaves and triphone (Tri2) modeling with 600 senones and 2500 leaves is performed. A minimum WER of 2.22% is achieved by the MFCC features when combined with the delta and double-delta features. Further, using 700 tied states and 3000 cluster leaves, a linear discriminant analysis with maximum likelihood linear transformation (LDA-MLLT) (tri3) is applied to the features, which is subsequently modeled using discriminative approaches, yielding 2.13% of the WER with the boosted feature-space MMI, and a relative improvement of 4% is observed in the discriminative training. These baseline results are compared with the 3-fold, 5-fold, and 7-fold speed-perturbated data, as shown in Figure 4a. The percentage error in the MMI, bMMI, fMMI, and fbMMI decreases significantly as the number of folds accumulates, as depicted in Figure 4b. When compared to the MMI and bMMI, the recognition performance of the fMMI and fbMMI is enhanced at a 7-fold augment, with an RI of 11.06% and 3.7%, respectively.

### 6.2. Performance Analysis of Proposed Models on Spontaneous Speech Task

The GMM-HMM acoustic model uses the same composition of Gaussians, tied states, and cluster leaves where Tri1 and Tri2 have the least WER compared to the fMMI and fbMMI, because the models are trained with relatively minimal data in the spontaneous speech task without speed perturbation. Because of their L2 [46] proficiency, children tend to include long pauses, repetition of words, and speech crossovers in spontaneous speech tasks, which indeed results in a performance drop-off in ASR when compared to reading speech proficiency. Long pauses can be diminished using speed-based data augmentation, and the recognition performance of feature discriminative techniques can be optimized.

**Figure 5:**
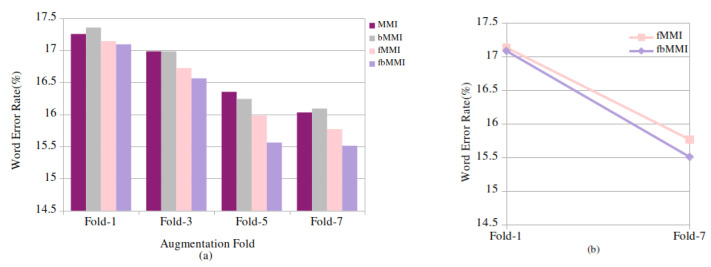
Spontaneous speech: (**a**) word error rate (%) for baseline (fold-1) and synthetic (fold-3, 5, 7) data on discriminative techniques and (**b**) (%) relative WER improvement on fMMI, fbMMI for baseline and 7-fold speed-perturbated data.

After the augmentation, 0.67 h of baseline training data is 7-fold perturbated to 4.95 h, resulting in a decrease in the WER from 17.14% to 15.77% in the fMMI and 17.09% to 15.51% in the fbMMI, respectively, as shown in Figure 5a. A relative improvement of 7.99% and 9.24% in the fMMI and fbMMI, respectively, is achieved in spontaneous speech, as depicted in Figure 5b.

### 6.3. Performance Analysis of Proposed Models on Combined Speech Task

With regard to speed perturbation-based augmentation, there is indeed a significant variation in the performance of the ASR on the read speech and spontaneous speech of non-native children, as shown in the preceding sections. However, both the read and spontaneous speech datasets are merged and explored with the proposed models in order to make the ASR a generic model for any type of speech.

**Figure 6:**
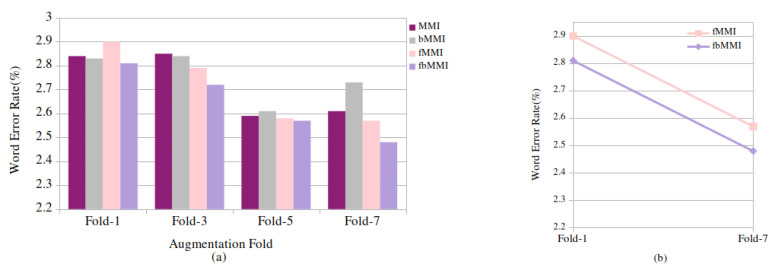
Combined speech: (**a**) word error rate (%) for baseline (fold-1) and synthetic (fold-3, 5, 7) data on discriminative techniques and (**b**) (%) relative WER improvement on fMMI, fbMMI for baseline and 7-fold speed-perturbated data.

With a 7-fold data augmentation, 2.65 h of training data is increased to approximately 20 h in combined speech. Alongside, the suggested models are evaluated on 0.67 h of orthogonal data without augmentation, and the best WER in the fbMMI is 2.81%. All the discriminative models performed similarly in this type of speech, as seen in Figure 6a. With a 5-fold speed perturbation, the MMI and bMMI models perform better than the 7-fold one; however, with the 5-fold and 7-fold augmentation, the fMMI and fbMMI models perform similarly. As shown in Figure 6b, the fMMI and fbMMI obtained a performance improvement of 11.37% and 11.74%, respectively, with the 7-fold speed perturbation, as presented in Table 5.

### 6.4. Comparative Analysis of Earlier State of the Art on Non-Native Children Speech Recognition

As reported in Table 6, the majority of non-native children’s speech recognition research has centered on languages such as Italian, German, Chinese, Swedish, and others, but ASR for Indian children who speak Telugu as their first language and English as a second has yet to be examined. In comparison to previous state-of-the-art models, this paper is a forerunner in addressing the issues faced by L2 learners while using ASR systems and has therefore developed a discriminatively trained non-native children’s ASR.

## 7. Conclusions

In this paper, a corpus of non-native children who are acquiring a second language such as English is being collected for a children’s speech recognition system and analyzed in a limited-data scenario. The speed perturbation-based data augmentation is induced with various folds (1, 3, 5, and 7) on the original data to handle the data scarcity. Through a comprehensive set of feature discriminative training experiments, the combined use of read speech and spontaneous speech has demonstrated the promising performance of non-native children’s English speech ASR. The results revealed that the system training-pooled 7-fold speed perturbation-based data augmentation outperformed the baseline models (1-fold) with relative improvements of 11.37% and 11.74% with the fMMI and fbMMI, respectively. The performance of the non-native children’s ASR may be significantly improved in the near future by developing more advanced DNN-HMM or end-to-end ASR systems and also other resource-poor ASR applications.

## Figures and Tables

**Table 1:** Details of non-native children speech corpora.

Baseline		Utterances	Total Words	Unique Words	Duration (hours)
Read Speech	Train	1585	18,154	155	1.82
	Test	588	6769	123	0.65
Spontaneous Speech	Train	569	6906	131	0.67
	Test	156	1896	82	0.18
Combined Speech	Train	2274	26,443	256	2.65
	Test	624	7286	183	0.67

**Table 2:** Examples of disfluency in non-native children speech corpus.

Type of Disfluency	Example
Word Repetitions	this is a umbrella ***it is it is*** useful when it rains
Word Fragments	there was a ***sa- sad*** dog he did not have any friend
Intra-Word Switching	everyone in my ***schooluu*** likes icecream
Hesitations	on his way home he ***hmm*** he crossed a river and saw another dog
Ungrammatical Words	this is a pencil we will use it when ***when we should write*** our home works

**Table 3:** Audio augmented data of children training set with different speed perturbation factors. Different number of hours (#Hrs) and the respective utterances (#Tokens) for training are used.

Datasets	Fold	Perturb. Factors	#Hrs	#Tokens
Read Speech	1	Baseline	1.82	1.58 K
	3	0.9, 1.0, 1.1	5.51	4.75 K
	5	0.8, 0.9, 1.0, 1.1, 1.2	9.32	7.92 K
	7	0.8, 0.85, 0.9, 0.95, 1.0, 1.1, 1.15	13.46	11.1 K
Spontaneous Speech	1	Baseline	0.67	0.56 K
	3	0.9, 1.0, 1.1	2.03	1.7 K
	5	0.8, 0.9, 1.0, 1.1, 1.2	3.43	2.84 K
	7	0.8, 0.85, 0.9, 0.95, 1.0, 1.1, 1.15	4.95	3.98 K
Combined Speech	1	Baseline	2.65	2.27 K
	3	0.9, 1.0, 1.1	8	6.82 K
	5	0.8, 0.9, 1.0, 1.1, 1.2	13.53	11.37 K
	7	0.8, 0.85, 0.9, 0.95, 1.0, 1.1, 1.15	19.53	15.9 K

**Table 4:** Experimental results of acoustic and discriminative models in %WER for original and synthetic data with different styles of children’s speech.

Type of Speech	Fold	Acoustic Models				Discriminative Models			
		Mono	Tri1	Tri2	Tri3	MMI	bMMI	fMMI	fbMMI
Read Speech	1	2.87	2.30	2.22	2.66	2.39	2.42	2.26	2.13
	3	2.60	2.67	2.22	2.25	2.11	2.20	2.07	2.07
	5	2.93	2.35	2.07	2.33	2.28	2.26	2.06	2.05
	7	2.90	2.16	2.06	2.42	2.16	2.33	2.01	2.05
Spontaneous Speech	1	19.51	15.82	16.17	17.25	17.25	17.35	17.14	17.09
	3	22.73	18.67	16.09	17.09	16.98	16.98	16.72	16.56
	5	21.78	16.77	16.51	15.98	16.35	16.24	15.98	15.56
	7	21.89	16.24	15.55	15.82	16.03	16.09	15.77	15.51
Combined Speech	1	3.43	2.85	2.96	3.03	2.84	2.83	2.90	2.81
	3	3.46	2.99	2.68	2.72	2.85	2.84	2.79	2.72
	5	3.69	2.83	2.64	2.92	2.59	2.61	2.58	2.57
	7	3.72	2.85	2.90	2.79	2.61	2.73	2.57	2.48

**Table 5:** Comparison of baseline and speed perturbation on different types of children’s speech.

Type of Speech	Fold	#Hrs	WER (%)		Rel. Improvement (%)	
			fMMI	fbMMI	fMMI	fbMMI
Read Speech	1	1.82	2.26	2.13	11.06	3.7
	7	13.46	2.01	2.05		
Spontaneous Speech	1	0.67	17.14	17.09	7.99	9.24
	7	4.95	15.77	15.51		
Combined Speech	1	2.65	2.90	2.81	11.37	11.74
	7	19.53	2.57	2.48		

**Table 6:** Comparative analysis of earlier state-of-the-art results.

Year/ Author	Augmentation Type	Dataset Type	Front-End Approach	State-of-the-Art Model	Performance
2017 [20]	No augmentation	English read, picture narration, and spontaneous speech (11–15 years) from native Arabic, Chinese, French, German, and many other children speaking English	MFCC	BiLSTM-RNN	WER of 13.4% is obtained
2018 [18]	No augmentation	Italian, German, English, and Swedish children aged 9–10 years	MFCC	DNN	Non-native adaption was used in transfer learning and results in 14.2% WER for Italian and 15% for German speaking English
2020 [21]	Prosody-based augmentation, spectrogram augmentation	TLT non-native corpus— English read speech from native Italian children (9–16 years)	MFCC	TDNN+BiLSTM +VTLN	WER of 18.71% with spectrogram augmentation
2020 [22]	Speed perturbation, spectrogram perturbation	TLT non-native corpus— English read speech from native Italian children (9–16 years)	MFCC+ i vectors, CMVN, VTLN	TDNN-F, CNN-TDNN-F	WER is 17.59% with semi-supervised learning
2020 [23]	Spectrogram augmentation	TLT non-native English, German, and Italian children corpus—spontaneous speech (9–16 years)	MFCC	TDNN-F+LSTM	WER is 15.7% by combining all systems independent of grade
2020 [24]	Speed perturbation, room impulse response (RIR), babble noise, non-speech noise	OGI, MyST, CU, CMU (5–16 years) read and spontaneous speech	MFCC	GMM-HMM CNN+TDNN-F	WER is 16.59 with min. Bayes-risk decoding
2020 [25]	Pitch, speed, volume, tempo, reverberation perturbations	SLT Mandarin children read and conversational speech (4–16 years)	MFCC	CNN-TDNN-F, EspNet	CER is 16.48% by combining all types of perturbations
2021 [47]	Data augmentation, time-scale modification	Clean adult’s speech WSJCAM0 (train data) Noisy children’s speech PF-STAR (test data)	MFCC	DNN-HMM	WER is 14.88% by all types of data augmentation in combined system
Proposed work	Speed perturbation- 3Way, 5Way, and 7Way	7–12 years of English read and spontaneous speech from native Indian (Telugu) children	MFCC CMVN	GMM-HMM MMI, bMMI, fMMI, fbMMI	WER for different styles of speech 2.01% (read), 15.51% (spontaneous), 2.48% (combined)

## Data Availability

The open-access data that support the findings of this study are made publicly available in the Kaggle repository, https://doi.org/10.34740/KAGGLE/DS/2160743, accessed on 9 May 2022. More details about the data collection are given in Section 3.
